# Culture, diversity and inclusion: a survey of British Hip Society members

**DOI:** 10.1136/bmjoq-2023-002432

**Published:** 2023-10-13

**Authors:** K Dayananda, K Gill, A Grove, J Parr, S Hook, J R Howell, J Maggs

**Affiliations:** 1 Trauma & Orthopaedic Surgery Welsh Rotation, Morriston Hospital, Swansea, UK; 2 Trauma and Orthopaedic Surgery, The Royal Surrey County Hospital NHS Trust, Guildford, UK; 3 Warwick Medical School, University of Warwick, Coventry, UK; 4 Trauma and Orthopaedic Surgery, Portsmouth Hospitals University NHS Trust, Portsmouth, UK; 5 Trauma and Orthopaedic Surgery, Royal Devon and Exeter Hospital, Exeter, UK; 6 Trauma and Orthopaedic Surgery, Torbay Hospital, Torquay, UK

**Keywords:** Organizational Culture, Emotional Intelligence, Staff Development

## Abstract

**Aim:**

To explore the perceptions and experiences of members of the British Hip Society (BHS) as they relate to culture, diversity and inclusion in the professional sphere.

**Method:**

BHS members participated in an anonymised online survey in 2021. Quantitative and qualitative data were collected on demographics, professional experiences and perceptions of workplace culture. Members provided suggestions for improving working culture and supporting inclusivity.

**Results:**

A 45% response rate (n=217) was achieved. Most respondents were male consultant surgeons, of white ethnicity. Almost a quarter of respondents reported experiencing barriers to career progression within the hip subspecialty. Experience of barriers was more common among women and those of non-white ethnicity. Several members experienced an elitist, exclusive culture in the BHS which is closed to outsiders. Thematic analysis of textual data revealed narratives which portray the perception of the society as a closed-door society, and described a clique culture in orthopaedics, and the pervasiveness of discrimination and banter.

**Conclusion:**

We found that barriers to inclusion and diversity exist within the professional society. Exploring the narratives around these has informed strategies to overcome them and has shaped future BHS initiatives. To ensure our patients receive the best possible surgical care, it is vital that those with the skills and expertise to deliver it, are supported by the Society and feel a sense of belonging and representation.

**WHAT IS ALREADY KNOWN ON THIS TOPIC**

- Best surgical care should be delivered by diverse teams, yet the British Hip Society was uncertain of the composition of their membership. We found it has a homogeneous professional population.

**WHAT THIS STUDY ADDS**

- The study provides a baseline demographic profile of members of the British Hip Society. It identified the barriers to inclusion and diversity which exist within the professional society.

**HOW THIS STUDY MIGHT AFFECT RESEARCH, PRACTICE OR POLICY**

- Further in-depth exploration of the narratives around inclusion and diversity in surgery could help to inform strategies to overcome barriers and shape future initiatives across the specialty.

## Introduction

Most literature exploring culture and diversity in surgery focuses on the under representation of women. In trauma and orthopaedics, just 7% of consultants are women,^1 2^ despite a more than 50% intake of women into UK medical schools for over 20 years. Studies examining ethnicity in surgery are emerging, but very little research has examined issues faced by doctors and trainees from overseas or specialty and associate specialist (SAS) grade doctors. We found no research which explored the impact of surgeons’ social class, disability and sexual orientation on their perceptions and experiences of surgery. Data from the British Orthopaedic Association and the Royal College of Surgeon England suggest that surgery in general, is neither attracting nor retaining a diverse workforce,^2 3^ though the reasons for this are poorly understood.^3^

The British Hip Society (BHS) in the UK was founded in 1989 to support training, education and research, thereby promoting the best care for patients with hip-related conditions. Our intention is to welcome specialist hip surgeons from all backgrounds and at all career stages. We recognise, however, that most of the members of our professional society appear to be British, male and white and, therefore, we need to encourage diversity. We believe that a surgical subspecialty that has a reputation for valuing diversity will attract and retain talent, increase innovation and perpetuate an enviable esteem internationally. The aim of this study was to capture the baseline demographics of our society, and to explore the perceptions and experiences of our members as they relate to culture, diversity and inclusion in the professional sphere.

## Methods

A questionnaire was codeveloped by the BHS Culture and Diversity Committee and an independent research company. This was distributed to BHS members using a secure, anonymised online platform. Responses were processed by the independent research company to ensure confidentiality and anonymity of responses. We were provided aggregate, anonymised demographic responses, presented by subgroup.

We asked members about their age, gender, ethnicity, disabilities, sexual orientation, religious identity, marital status and job role. We also asked about their personal experience of barriers to career progression relating to equality and diversity, and their experiences of exclusion from social and professional conversations. Members were asked to outline what they thought the society could do to encourage a greater level of inclusion in social and professional interactions between members (online supplemental appendix 1 provides an example survey including all 40 questions).

10.1136/bmjoq-2023-002432.supp1 Supplementary data

We present the demographic data separately from the open-ended survey responses, to ensure that anonymity of respondents is maintained. We draw on comparisons to National Health Service (NHS) demographic data to contextualise our findings.^1^ We used Microsoft Excel (Microsoft 2022) to sort and analyse the numeric data by frequency. Free text responses were processed in NVivo (QSR International 2020). Responses from the open-ended questions were analysed thematically to identify, and interpret patterns of meaning (i.e., the themes) within our survey responses.^4^

## Results

Our survey response rate was 45% (n=217) which we consider reasonable and consistent with survey-based research.^5^

### Demographics of professional membership

**Figure 1:**
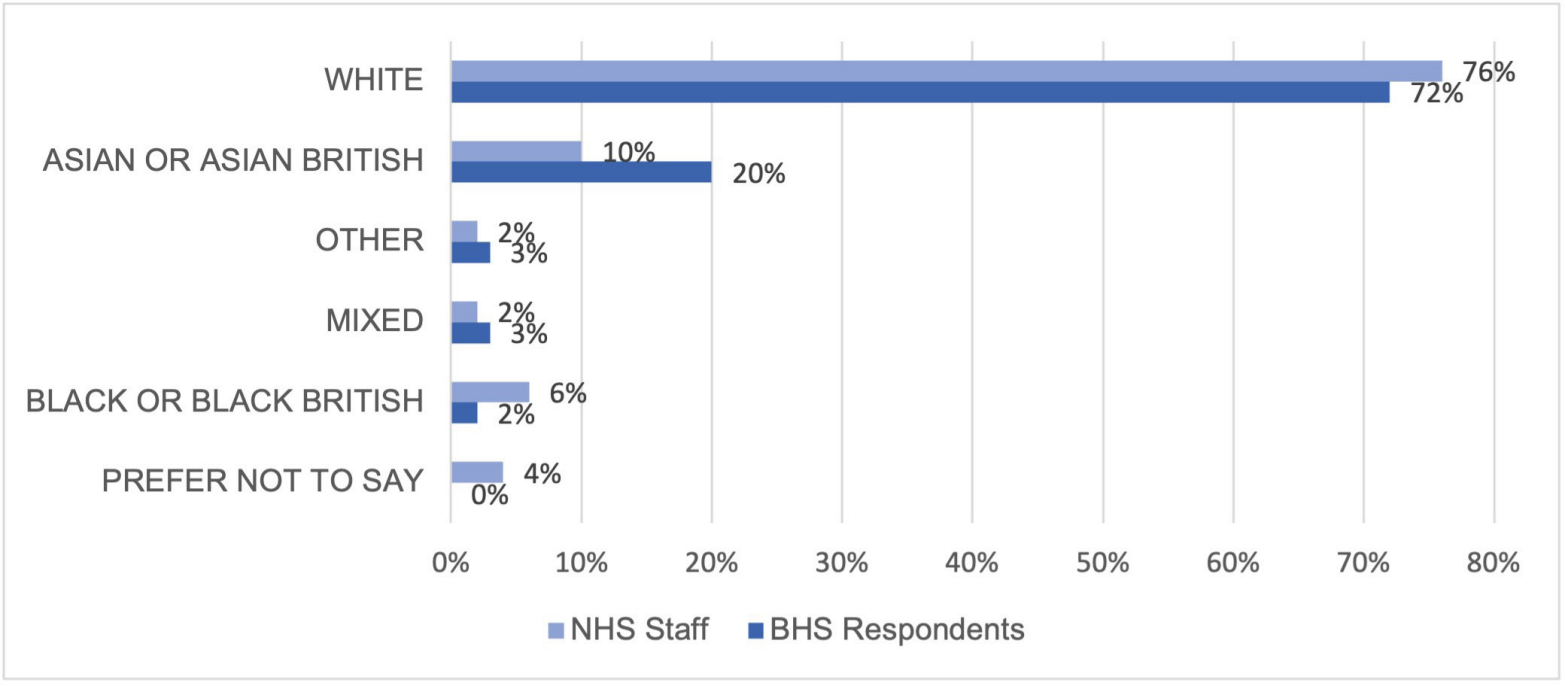
Ethnicity of BHS survey respondents compared with National Health Service (NHS) workforce. (BHS) British Hip Society.

- Age: Survey respondents tended to be older than the average NHS consultant body, with most respondents aged 45–54 (43% vs 40%, respectively). We had a greater number of respondents in the 65+ age group (8% vs 3%) and a lower number of respondents in the 35–44 age group compared with the NHS consultant body (25% vs 36%).
- Grade: 88% of respondents were consultants, 5% were post certificate of completion of training (CCT) fellows, 4% were specialty trainees, 1% were SAS surgeons, and 1% scientists.
- Gender: 94% of our respondents were male, 5% were female, 1% preferred not to say.
- Ethnicity: 72% of respondents described their ethnicity as White, 20% as Asian or Asian British and 2% as Black or Black British. Our respondents included a greater proportion of those of Asian or Asian British ethnicity (20% vs 10%) and mixed ethnicity (3% vs 2%) compared with the NHS workforce. We found a lower proportion of those of Black or Black British ethnicity, (2% vs 6%) and White ethnicity (72% vs 76%) compared with the NHS workforce (see figure 1).
- Disability: A small proportion of surgeons reported that they had a disability (4%), which is comparable to the wider NHS workforce (5%).
- Marital status: Most of the respondents to our survey were married (88%).
- Sexual orientation: 95% of respondents stated they were heterosexual, with 2% self-identifying a different sexual orientation, 3% stated that they preferred not to respond. For comparison, the data for the wider NHS workforce describe 70% as heterosexual, 3% as Lesbian, Gay, Bisexual, Transgender, Queer, and Intersex+ (LGBTQI+) and 27% who prefer not to say.
- Religion: 53% stated that they were Christian, 24% said they had no-religion or were atheist, 15% identified as Hindu, 4% Muslim, 2% other and 1% Jewish. When compared with the wider NHS workforce, our sample has higher rates of those practising Hinduism and Christianity.

Perceptions and experiences of our members as they relate to culture, diversity and inclusion.

### Barriers experienced in the past

We asked members of the society ‘Have you personally experienced any barriers relating to equality or diversity in your progression within the Hip subspeciality of Trauma & Orthopaedics (T&O) within the past 5 years?’

**Figure 2:**
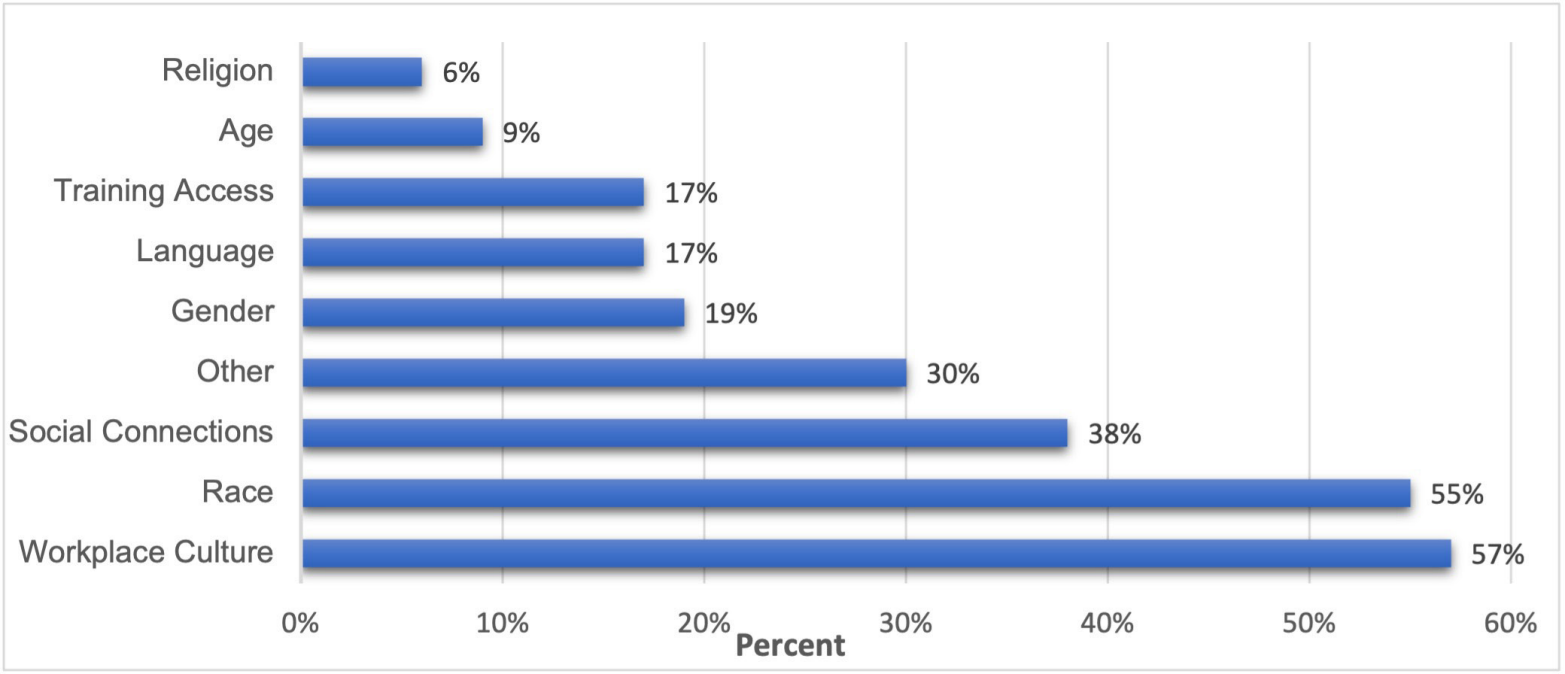
Equality and diversity barriers experienced by respondents who answered ‘yes’.

Twenty-three per cent of our respondents answered ‘yes’ and 77% said ‘no’. Of those people who reported experiencing barriers, most were related to workplace culture (57%) and race (55%), followed by social connections (38%) and gender (19%) (see figure 2).

Women were more likely to report having experienced barriers compared with other gender groups; 90% of women and 19% of men reported experiencing barriers.

**Figure 3:**
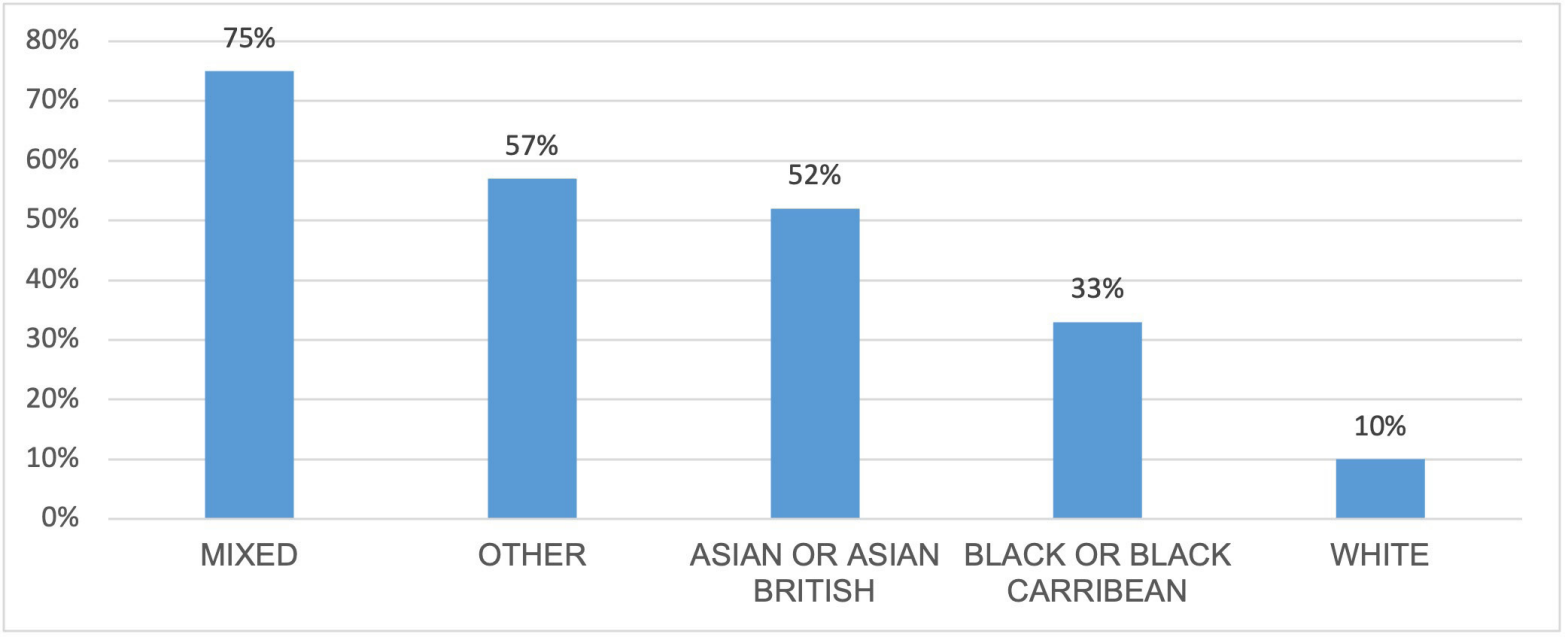
BHS survey: ethnic background of those reporting experience of barriers. BHS, British Hip Society.

People who were not White also reported higher rates of having experienced barriers: 75% of mixed-race surgeons reported experience of barriers, as did 52% of Asian or Asian British surgeons, 33% of Black or Black Caribbean surgeons and 10% of White surgeons (see figure 3).

**Figure 4:**
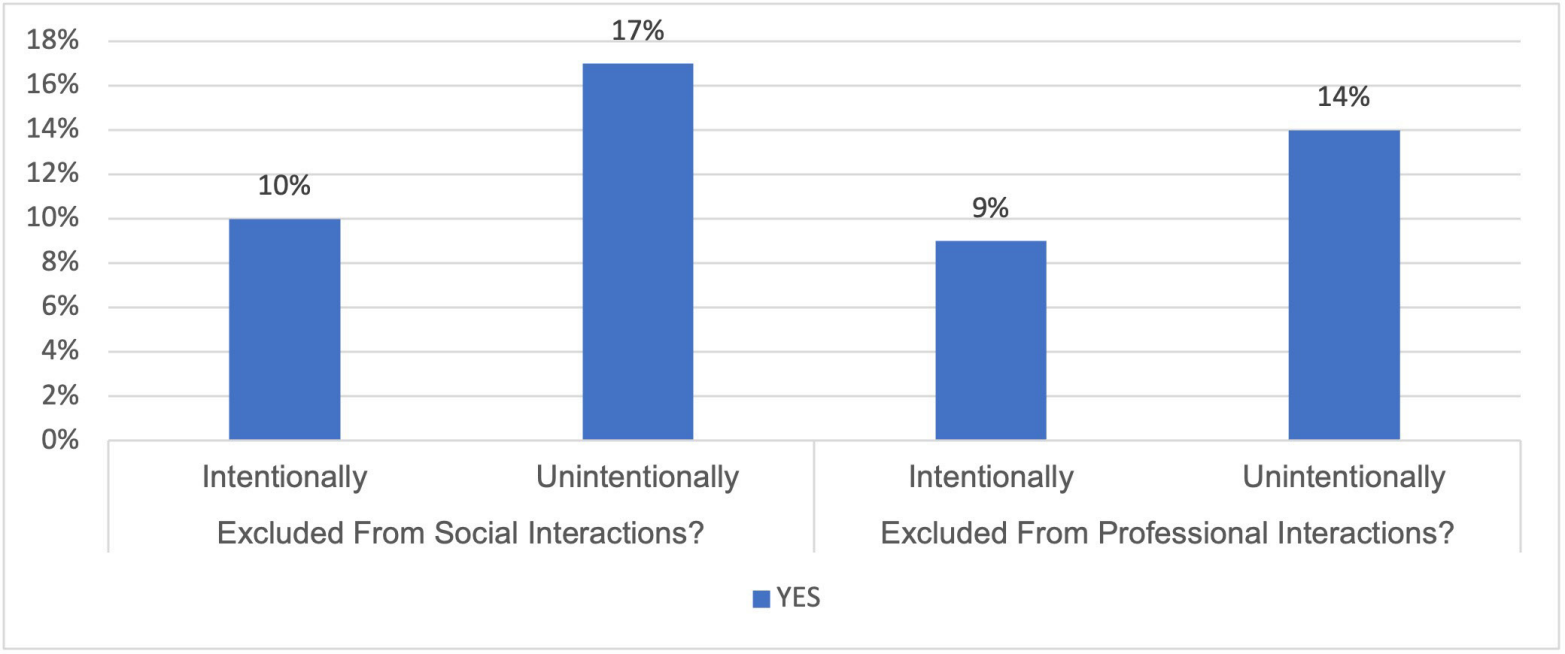
Experience and perception of: social interactions (left) and professional interactions (right).

Thirty-eight per cent of society members said they had experienced barriers related to social connections within the last 5 years. Of those who reported experiencing exclusion from social interactions, 10% described this as intentional exclusion. Of those who said they had experienced professional exclusion, 9% described this as intentional exclusion (see figure 4).

### Narrative findings

Three prominent themes were derived from analysis of free-text responses: (1) a perceived elitist society, (2) the clique culture in orthopaedics and (3) the pervasiveness of discrimination and banter. Theme 1 refers specifically to the Society, themes 2 and 3 reflect the wider profession of trauma and orthopaedic surgery in the UK.

#### A perceived elitist society

There was a perception among responders that the professional society was a small and exclusive group, which is closed to outsiders. One surgeon described the need to ‘break’ into the society: ‘*The feeling is that the top level is a ‘closed-door group’ making it difficult for the average person to break in*.’ Other surgeons described the BHS as a ‘*bit of a boys club*.’ Some respondents described what was summarised by one surgeon as ‘*a feeling of London centric elitism*.’ Another explained how ‘*it is rare for someone from a provincial small DGH [district general hospital] to get a look in’*. The importance of where you work, and your connections, to broker entry were highlighted by respondents. One surgeon described the location of work as important to gain entry to the exclusive group: ‘*[if you] Belong to smaller hospital, there is imbalance in the partnership with all key roles taken by the larger organisation[s].’* Surgeons described that those behind the ‘closed door’ at the top of the organisation didn’t always recognise alternative aspects of the profession which are important to surgeons and patients: ‘*There are other qualities that someone can bring to the Society like being a good trainer/teacher, which previously I felt wasn’t sufficiently valued.’* It was perceived by some respondents, that the executive committee of the Society lacked diversity: ‘*The BHS executive does not represent the diversity of its membership in terms of age, ethnicity and cultural differences*.’

The perception of the Society as an elite society was experienced acutely at meetings and in conference environments, with many surgeons describing a lack of confidence in attending these events. This was particularly prominent when surgeons were new to the profession, for example, new trainees or international doctors working in the NHS. Several of our respondents gave examples of discrimination which was disguised as ‘*banter’*. Surgeons described discrimination that resulted from their ‘*accent and pronunciation of some words’*, which at work became a ‘*topic for banter’*. There was a suggestion that ‘*without realising it, talk often falls to male banter*.’ Personal and family culture can be linked to religious beliefs and surgeons reported the large impact ‘*blasé comments about religion and culture’* can have on a person’s identity, which goes unappreciated by others. One surgeon suggested that the Society should initiate ‘*ground rules’* (eg, no tolerance for discrimination) at their conferences to make society meetings feel like less intimidating spaces.

#### Clique culture in orthopaedics

One of the most prominent words used to describe orthopaedics in our survey response was ‘*clique’*. References to clique cultures appeared to relate to characteristic stereotypes within the profession: ‘*The senior culture in orthopaedics is still very dominated by WASP [White Anglo-Saxon Protestants] ex-rugby players who only trust their own’.* Many surgeons described negative impacts of cliques. Some respondents suggested that professional appointments, were made in an unfair way, using descriptions such as the ‘*mafia’* and ‘*undemocratic’* to describe appointment of surgeons into key roles across some hospitals. Others described the ‘*difficulty in developing networks in some situations as not part of a drinking circle of senior surgeons’*. Some respondents described challenges during training, their employers’ attitude and the hierarchical nature of orthopaedics as barriers to professional success and opportunities. A number of younger surgeons described similar feelings of being ‘*overlooked by senior surgeons who dismissed my opinions based on the fact they were older than me or more important because of their position’*.

We found repeated descriptions of cultural beliefs around physical strength, and the need to be physically strong as a requirement for entry into the surgical clique. One surgeon portrayed orthopaedics and hip surgery as ‘*still regarded as a physically challenging job and hence there are issues regarding gender, and physical attributes’* other respondents described perceptions that ‘*girls are not strong enough to be arthroplasty surgeons’*. One surgeon described the ‘*difficulty seeing myself as a consultant in a man’s world…never having had a female trainer’*. Another states: ‘*several trainers discouraged me from a subspecialty in Hip Surgery as I was a woman,’* and ‘*although difficult to prove, there have also been incidents where I have felt less respected than male colleagues with regards to my specialty choice’*.

#### Pervasiveness of discrimination and banter

Respondents highlighted myriad ways in which discrimination affects both trauma and orthopaedics in general, and hip surgery in particular. Our findings suggest that because orthopaedics is perceived an elitist culture, schooling and family background can impact career progression. References were made to the expense of surgical training and difficulties in sustaining a career without financial support, which was applicable only to certain members of the profession. One surgeon described how: ‘*Career progression is expensive. Courses, meetings, conferences, fellowships are paid with own money’* and another explains that ‘*achieving [a] flexible / part-time contract to support my family was challenging’*. Another respondent described how ‘*it is easier to pass exams, progress career if you have family wealth’*.

However, it is not just the cost and (in)flexibility of training that was problematic for our respondents. There was a perceived importance attached to social class and status, which resulted in discrimination in the workplace. A respondent reflected how ‘*workplace culture is one of white middle class males, if you don't meet these criteria, your opinion and input is not valued, respected or asked for’*. Insight from a respondent who attended comprehensive school illuminate perceived social class barriers in orthopaedic surgery: ‘*As a comprehensive school educated doctor then progressing through surgical training and into consultancy at most junctures there was elitism and derisory opinions on those of us who weren’t ‘born into’ medicine or money.’*

Examples of discrimination on the grounds of gender and race were also reported. One respondent described ‘*male colleagues who do not think women should do revisions*.’ One surgeon stated that as ‘*a BAME doctor [I have to] work harder to reap the same benefits as a white counterpart*.’ Others described witnessing discrimination in the workplace: ‘*I have witnessed surgeons being racist and sexist behind closed doors and overtly.’* Systemic racism across healthcare was also highlighted, one surgeon suggested that ‘*GPs are more likely to refer nicer patients directly to white sounding consultants on named basis than non-white*.’ In our survey, we received only one response pertaining to sexual orientation. This surgeon described how they ‘*felt privileged’* because they were a ‘*white, heterosexual male’* and therefore, a member of the professional majority.

A few responses proposed that minority groups may benefit, rather than be disadvantaged, by their characteristics. One respondent described their views on the impact on gender in the profession: ‘*My view is that females, especially those who are attractive, are at a positive advantage when attempting to get funding / fellowships / consultant posts when there is an all-male panel / male dominant panel appointing*.’ This view was echoed by other surgeons who said that ‘*the white male is under attack’* and that ‘*I have diverse views and yet my phenotype is taken as the only issue’*. Other surgeons described a general perception of ‘*unconscious bias towards non-white males in orthopaedics as a whole’*. One surgeon described that their ‘*department have openly expressed feelings of pressure to appoint candidates from minority groups’*.

## Discussion

Our study aimed to explore the perceptions and experiences of members of our professional surgical society as they relate to culture, diversity and inclusion in the professional sphere. Capturing the demographic profile of our membership was important so that we could identify under-represented groups and consider how we may be better able to attract and support them. The small number of female and Black or Black British respondents was a particularly stark yet unsurprising finding.

The respondents to our survey are members of the society and they include trainees, post-CCT fellows, consultants, non-consultant specialty surgeons and scientists. Missing from this study, however, are the voices of those who at one time may have considered a career in hip surgery, but ultimately opted for a different path, perhaps resulting from the equality and diversity barriers we have identified in our survey.

Understanding our members’ perceptions and experiences of barriers to their progression (in the past 5 years), relating to equality or diversity, and their beliefs around barriers to their further progression within the subspecialty, is essential to discovering the ways to tackle and overcome barriers in future. There is evidence from diversity science that diverse teams improve decision making and achieve better outcomes when compared with homogeneous teams.^6 7^ This phenomenon is more apparent when teams are engaged in complex tasks involving social interaction, such as when undertaking surgery and perioperative care.^8^ Improving the diversity of our profession is also vital for our patients. There is a growing body of evidence to suggest that a more diverse medical workforce will improve access to healthcare for patients from marginalised and under-represented groups.^9 10^

Three powerful themes emerged from thematic analysis of narrative responses: the perception that the BHS is an elitist society, that there is a clique-culture within the orthopaedic profession, and that discrimination and banter is pervasive among the wider profession. In our study, we provide data detailing the perceptions and experiences of surgeons who suggest that discrimination remains rife in orthopaedics, which is of huge concern.^11^ The Equality Act 2010 aims to provide protection from discrimination, harassment, and victimisation based on age, disability, gender reassignment, marriage and civil partnership, race, religion or belief, sex and sexual orientation.^11^ However, in our study, we found several examples of perceived discrimination across many of these protected characteristics, with ethnicity and gender being most prevalent. We found female surgeons who described being actively discouraged from the specialty. The quarter of respondents who reported feelings or perceptions of social or professional exclusion within the profession only compound the issues of exclusivity and discrimination in orthopaedics.

### Strengths, limitations and future work

This is the first survey to examine the culture of the BHS. We must stress that at times our findings appeared to reflect the experiences and perceptions of orthopaedics generally, rather than hip surgeons specifically. However, this provides useful information about diversity, equality and inclusion in the wider profession and demonstrates that broad scale changes are needed, not just within the BHS. Although care was taken to distinguish which element participants were referring to (BHS or the wider profession), this was not always an easy distinction to make when interpreting our findings.

A significant limitation of our study is that respondents overall were unrepresentative of the groups most affected by issues of diversity, equality and inclusion, in particular a range of ethnic groups, younger surgeons and women. When reading our findings, it should be born in mind that groups most affected by issues of equality and diversity were the minority of responders, which lends weight to taking a qualitative approach, but suggests more in-depth investigation is required. It is important to highlight that we have not reported all the survey items in this paper (see online supplemental appendix 1). We excluded those responses which were informative to Society as part of their service evaluation; for example, ‘*How would you rate the British Hip Society on encouraging wider social engagement between members?’*. The responses to these questions were included in the final report which was submitted to the BHS Committee in help inform their improvement activities.

Our survey provides a snapshot of the demographic profile of members of the BHS and T&O profession, but total numbers are difficult to establish. While we collected data on gender and ethnicity, other protected characteristics, such as disability, sexual orientation and religion were lacking. More detailed metrics to assess demographic profiles are required to provide a truly representative picture of the composition of our workforce. When doing this, we must acknowledge the importance of intersectionality. It is important to recognise that surgeons are people and have diverse identities and distinctive experiences of discrimination that may not be captured in relatively simple surveys.

As our survey was self-reported, there is a potential for responder bias; and some people may have felt more compelled to engage than others. It is, therefore, not possible to extrapolate the demographic characteristics of responders to the entire membership. A wide variety of views were received, but our findings suggests that we may be missing the perceptions and experiences of some groups of people, or indeed potentially that those facing these issues were more inclined to respond to our survey. Taking account of bias introduced by our cross-sectional self-report survey design, we were surprised to see few or no comments relating to less than full time working, sexual orientation, disability, gender reassignment, marriage or civil partnership, pregnancy and family leave. The issue of work location was not asked in the survey and is something that warrants further attention to explore the relationship between workplace and professional and or protected characteristics. An area for future research is to explore whether surgeons and trainees have grappled with some of these issues and chose to leave the subspecialty. It is important to capture and learn from those people who have faced challenges and barriers that, at the time, could not be resolved.

## Conclusion

The BHS membership lacks diversity with some groups, most notably female, and Black or Black British surgeons, being under-represented. In our membership survey, there were pervasive feelings that the professional society is an elitist, which can be perceived as exclusive and excluding. More widely, trauma and orthopaedics was described as having a clique-culture, with discriminatory behaviour being commonplace, and frequently minimised as banter. Our Society recognises that provision of optimum patient care relies on training and supporting the best surgeons. If our profession is to remain competitive, we need to attract, include, and retain more diverse surgeons who better reflect the patients that we serve. As an organisation, we are committed to doing this, and through the BHS Culture and Diversity Committee, we have already implemented several initiatives to start changing culture for the better, although we recognise that we have a long way to go.

## Data Availability

Data may be obtained from a third party and are not publicly available. The data that support the findings of this study are available from the British Hip Society (BHS). Restrictions apply to the availability of these data, which were used under license for this study. Data are available AG with the permission of BHS.
