# Supplementary material for: Culture, diversity and inclusion: a survey of British Hip Society members
Source: BMJ Open Qual. 2023 Oct 13;12(4):e002432. doi: 10.1136/bmjoq-2023-002432 (PMC10582844; doi:10.1136/bmjoq-2023-002432)
Supplement: Supplementary data [file bmjoq-2023-002432supp001.pdf]

Dear Society Member,

Thank you for your interest in our survey which considers the very important issue of diversity and inclusion within our society. We hope that you will feel able to participate freely, safe in the knowledge that the survey is both voluntary and anonymous.

Sharing your feedback should take between 5 to 10 minutes of your time. You will see a few open text questions - we encourage you to share your experience, however if you prefer not to do so, please just write Nil in the relevant box instead.

Thank you for your support by completing the survey.

Demographics

This section of the survey asks for your responses to the standard NHS equality and diversity survey. We intend to compare the aggregated responses of the British Hip Society to wider NHS data to understand existing levels of demographic diversity and identify where improvements are necessary.

No individuals will be identifiable in any reporting.

1. **What year were you born?** *(to allow us to understand age diversity within the membership)*

2. **What is your ethnic group?**
   *Please select **one** of the below which best describes your ethnicity*

   Asian – Bangladeshi
   Asian – Chinese
   Asian – Indian
   Asian – Pakistani
   Asian – Other

   Black – African
   Black – Caribbean
   Black – Other

   Mixed – White / Asian
   Mixed – White / Black African
   Mixed – White / Black Caribbean
   Mixed – Other

   White – British Isles origin
   White – European origin
   White – Travelling Community
   White – Other

   Other – Arab
   Other – Please write in:

3. **Do you have a disability as defined by the equality act 2010**
   No
   Yes
   Prefer not to say

4. **How would you describe your gender?**

*Please select **one** of the below*
Female
Male
Prefer to self-describe: please write in
Prefer not to say

5. **What is your legal marital or civil partnership status?**
Divorced
Formerly in a registered civil partnership which is now dissolved
In a registered civil partnership
Married
Never married and never registered a civil partnership
Separated, but still in a registered civil partnership
Separated, but still legally married
Surviving partner from a registered civil partnership
Widowed
Prefer not to say

6. **What is your religion?**
*Please select **one** of the below*
Buddhist
Christian (including all denominations)
Hindu
Jewish
Muslim
None
Sikh
Other: please write in

7. **How would you describe your sexual orientation?**
*Please select **one** of the below*
Bisexual
Do not know
Gay or Lesbian
Heterosexual or Straight
Prefer to self-describe: please write in
Prefer not to say

<u>Career Experience</u>

This section of the survey aims to understand if you have experienced any barriers to progressing within the Hip subspeciality of Trauma & Orthopaedics.

8. **What position do you hold currently?**
*Please select **one** of the below*
Trainee
Training Interface Group (TIG) Fellowship
Post CCT Fellow
Consultant
Non-Consultant Specialty Surgeon
Scientist
Allied Health Professional

Have you personally experienced any barriers relating to equality or diversity in your progression within the Hip subspeciality of Trauma & Orthopaedics (T&O) within the past 5 years?

9. No, I have experienced no barriers to my progression relating to equality or diversity within T&O within the past 5 years
10. Yes, I have experienced barriers related to age within the last 5 years
11. Yes, I have experienced barriers related to gender within the last 5 years
12. Yes, I have experienced barriers related to language within the last 5 years
13. Yes, I have experienced barriers related to race within the last 5 years
14. Yes, I have experienced barriers related to religion within the last 5 years
15. Yes, I have experienced barriers related to social connections within the last 5 years
16. Yes, I have experienced barriers related to training access within the last 5 years
17. Yes, I have experienced barriers related to workplace culture within the last 5 years
18. Yes, I have experienced other barriers within the last 5 years

19. Would you like to elaborate on any barriers to progression you have experienced within the Hip subspeciality of Trauma & Orthopaedics?

Free text field

Do you believe there are any barriers relating to equality or diversity to your <u>further</u> progression within the Hip subspeciality of Trauma & Orthopaedics?

20. No, I perceive no further barriers to my progression relating to equality or diversity
21. Yes, I perceive there are barriers related to age
22. Yes, I perceive there are barriers related to gender
23. Yes, I perceive there are barriers related to language
24. Yes, I perceive there are barriers related to race
25. Yes, I perceive there are barriers related to religion
26. Yes, I perceive there are barriers related to social connections
27. Yes, I perceive there are barriers related to training access
28. Yes, I perceive there are barriers related to workplace culture
29. Yes, I perceive there are other barriers: please write in:

30. Would you like to elaborate on your <u>perception of barriers to further progression</u> within the Hip subspeciality of Trauma & Orthopaedics?

Free text field

<u>Perception of the British Hip Society</u>

This section of the survey aims to understand your current perception of the British Hip Survey, relating to its progress in developing a diverse and inclusive workplace culture.

31. How would you rate the British Hip Society on its <u>commitment to developing</u> an environment where people are encouraged to contribute, innovate and lead regardless of gender, ethnicity, sexual orientation or physical disability?

Weak	Below average	Average	Above Average	Strong

32. How would you rate the British Hip Society on its <u>progress towards</u> to developing an environment where people are encouraged to contribute, innovate and lead regardless of gender, ethnicity, sexual orientation or physical disability?

Poor	Below average	Average	Above Average	Excellent

33. **How would you rate the British Hip Society on its communication with members?**

Poor	Below average	Average	Above Average	Excellent

34. **How would you rate the British Hip Society on encouraging wider social engagement between members?**

Poor	Below average	Average	Above Average	Excellent

35. **Have you ever felt intentionally excluded from social conversations with British Hip Society colleagues (i.e. you were prevented from participating in the conversation)?**

    Free text

36. **Have you ever felt unintentionally excluded from social conversations with British Hip Society colleagues (i.e. you were included in the conversation but unable to participate)?**

    Free text

37. **What from your perspective would encourage greater social interaction and inclusion among British Hip Society members?**

    Free text

38. **Have you ever felt intentionally excluded from professional interaction with British Hip Society colleagues (i.e. you were prevented from participating in the conversation)?**

    Free text

39. **Have you ever felt unintentionally excluded from professional interaction with British Hip Society colleagues (i.e. you were included in the conversation but unable to participate)?**

    Free text

40. **What from your perspective would encourage greater professional interaction and inclusion among British Hip Society members?**

    Free text

Thank you for your responses to this questionnaire. Information will be collated anonymously with other responses by an independent research agency to produce data to assist our progress towards an inclusive and diverse workplace.
